# Associations of Indigenous language knowledge and physical, emotional, mental, and spiritual balance among First Nations living on reserve in British Columbia, Canada

**DOI:** 10.17269/s41997-025-01077-7

**Published:** 2025-07-01

**Authors:** Brandi Anne Berry, Nicole S. Berry, Marianne Ignace, Jeff Reading, Scott Venners

**Affiliations:** 1 Faculty of Health Sciences, Simon Fraser University, Burnaby, BC Canada; 2 Department of Linguistics and Department of First Nations Studies, Simon Fraser University, Burnaby, BC Canada; 3 British Columbia First Nations Health Authority Chair in Heart Health and Wellness, I-HEART Centre St. Paul’s Hospital, Providence Health Care, West Vancouver, BC Canada

**Keywords:** First Nations wellness, Language fluency, Cultural wellness, Mixed methods, British Columbia, Bien-être des Premières Nations, Maîtrise de la langue, Bien-être culturel, Méthodes mixtes, Colombie-britannique

## Abstract

**Objectives:**

A First Nations perspective on wellness includes physical, mental, emotional, and spiritual balance. Indigenous languages hold cultural knowledge and values that could promote wellness. Language learning is one way that Indigenous peoples may reclaim their cultural identity. We theorize that Indigenous language knowledge is one of multiple cultural activities causally downstream from Indigenous reclamation of culture among other causal precursors.

**Methods:**

Our analysis was informed by the results of qualitative interviews with ten Indigenous language learners. We conducted cross-sectional analysis of the First Nations Regional Health Survey (2015–2017) from adults living on First Nations reserves in British Columbia, Canada. Using logistic regression with adjustment for confounding, we estimated associations of Indigenous language knowledge with self-reported physical, mental, emotional, and spiritual balance.

**Results:**

In models adjusted for age and sex and compared to those with little or no fluency, among those with intermediate or fluent Indigenous language ability, the odds ratios (95% CI) of being in balance most or all of the time were 1.06 (0.79, 1.42) for physical balance, 1.23 (0.93, 1.62) for mental balance, 1.19 (0.90, 1.58) for emotional balance, and 1.57 (1.18, 2.10) for spiritual balance. In models adjusted for age, sex, and multiple cultural activities, these were 0.94 (0.69, 1.28); 1.05 (0.79, 1.41); 0.99 (0.73, 1.33); and 1.13 (0.82, 1.55) respectively.

**Conclusion:**

In age/sex-adjusted models, Indigenous language knowledge acted as a proxy for multiple cultural activities theoretically downstream from reclamation and promoters of cultural wellness. Our results are consistent with First Nations cultural activities promoting spiritual balance in this population.

**Supplementary Information:**

The online version contains supplementary material available at 10.17269/s41997-025-01077-7.

## Résumé

**Objectifs:**

Le point de vue des Premières Nations sur le bien-être inclut l’équilibre physique, mental, émotionnel et spirituel. Les langues autochtones contiennent des connaissances et des valeurs culturelles qui pourraient favoriser le bien-être. L’apprentissage des langues est une façon pour les peuples autochtones de se réapproprier leur identité culturelle. Nous postulons l’hypothèse que la connaissance des langues autochtones est l’une des plusieurs activités culturelles causalement en aval de la réappropriation autochtone de la culture, parmi d’autres précurseurs causals.

**Méthodes:**

Notre analyse se sert des résultats d’entrevues qualitatives menés auprès de dix Étudiant(es) de langues autochtones. Nous avons effectué une analyse transversale de ‘First Nations Regional Health Survey (2015–2017)’ auprès d’adultes vivant dans les réserves des Premières Nations en Colombie-Britannique, au Canada. Avec l’aide de méthodes de régression logistique, en ajustant pour les facteurs de confusion, nous avons évaluer les associations entre la connaissance des langues autochtones et l’équilibre physique, mental, émotionnel et spirituel auto-évaluées.

**Résultats:**

Dans les modèles ajustés en fonction de l’âge et du sexe et comparés à ceux qui avait peu ou aucune connaissance de la langue, parmi ceux qui avaient une maîtrise intermédiaire ou courante de la langue autochtone, les rapports de cotes (IC à 95%) d’être en équilibre la plupart du temps ou tout le temps étaient de 1,06 (0,79, 1,42).) pour l’équilibre physique, 1,23 (0,93, 1,62) pour l’équilibre mental, 1,19 (0,90, 1,58) pour l’équilibre émotionnel et 1,57 (1,18, 2,10) pour l’équilibre spirituel. Dans les modèles ajustés pour l’âge, le sexe et les multiples activités culturelles, ceux-ci étaient de 0,94 (0,69, 1,28); 1.05 (0,79, 1,41); 0,99 (0,73, 1,33); et 1,30 (0,82, 1,55) respectivement.

**Conclusion:**

Dans les modèles ajustés selon l’âge et le sexe, la connaissance des langues autochtones agissait comme un substitut à de multiples activités culturelles théoriquement en aval de la récupération et des promoteurs du bien-être culturel. Nos résultats sont cohérents avec les hypothèse que les activités culturelles des Premières Nations favorisant l’équilibre spirituel au sein de cette population.

## Background

Previous research has demonstrated that Indigenous language knowledge is associated with positive health outcomes including lower rates of diabetes (Oster et al., 2014) and rates of First Nations youth suicide (Chandler & Lalonde, 1998; Hallett et al., 2007, 2008). Substantial qualitative literature has described the importance of Indigenous languages to wellness and self-identity (Barker et al., 2017; Brown et al., 2012; Chew et al., 2015; Lee, 2009; T. L. McCarty et al., 2018; Taff et al., 2018; van Dijk, 2019; Whalen et al., 2016). There are 34 actively spoken First Nations languages in British Columbia (BC), each with programmes supporting learning and revitalization (First Peoples Cultural Council, 2022). This paper contributes to this literature by utilizing data from a survey developed by and for First Nations Peoples in what is known as Canada (First Nations Health Authority, 2019) and applying directed acyclic graphs for control of confounding (Hernán & Robins, 2024) to investigate the associations of Indigenous language knowledge with holistic measures of wellness.

Indigenous knowledge, wisdom, cultural understandings, and identity are embedded in Indigenous languages (Chew et al., 2015). The theoretical framework of this research positions language knowledge as one significant way among multiple cultural activities that Indigenous individuals might reclaim their identity. Reclamation of identity, in defiance of colonial policies, might be one way that participation in cultural activities can promote wellness (Barker et al., 2017; T. McCarty & Lee, 2014). Historical and contemporary colonial policies and practices have impacted language transmission in Canada, (Ignace, 2016) resulting in not only a loss of fluent language speakers, but also loss of culturally embedded knowledge (Brown et al., 2012; T. L. McCarty, 2019; Oster et al., 2014).

**Fig. 1:**
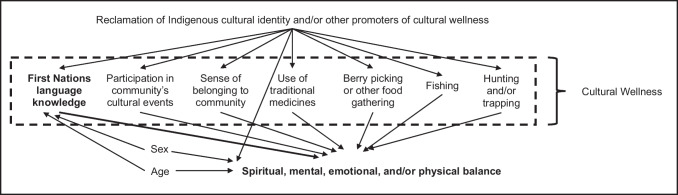
Directed acyclic graph depicting theoretical framework. Figure created in MS PowerPoint

Reclamation is one of many causal pathways by which a person may begin to engage in learning their language, or cultural activities writ large. Other antecedents to language learning are broad and may include a passion for learning a language, exposure to language in childhood, immersion programming, or a cultural responsibility to learn a language (Dunlop et al., 2018). We did not include these other causes of First Nations language knowledge in our directed acyclic graph (DAG, Fig. 1) because they are not necessary for completeness of the DAG. We mention them because they are important antecedents of language knowledge and learning that we do not want to omit from our narrative.

Reclamation in our theoretical framework is a highly individualized process by which a person reconnects to their Indigenous identity and culture, set in the context of long-term and ongoing colonial violence which has created structural inequities that effect Indigenous Peoples’ health, well-being, and self-determination. Other motivators of cultural wellness are outlined above, and there are likely many other factors beyond these that promote continuity of cultural wellness. In this framework, reclamation and other promoters of cultural wellness motivate (and thus occur prior to) more fulsome engagement in cultural activities. We envision all as causally upstream from participation in multiple cultural activities, including learning or maintaining one’s Indigenous language knowledge. Although some individuals may be involved in cultural activities prior to reclamation, we theorize that reclamation and other promoters of cultural wellness are common causal precursors to participation in multiple cultural activities when looking across the entire population in aggregate. This is supported by literature that either identifies reclamation as the inverse of colonization, which sets people towards a path of participating in cultural activities (including language) (Corntassel & Bryce, 2012; Hunter et al., 2006), or that the process of becoming more proficient in language encourages a more fulsome embodiment of Indigenous identity (Brown et al., 2012; Chew et al., 2015; Lee, 2009).

Our theoretical approach to measuring cultural wellness integrates two different, previous approaches. First, the number of cultural activities a person reports taking part in have been theorized to measure a latent construct of cultural wellness among First Nations living on reserves in what today is known as British Columbia, Canada (BC), by the First Nations Health Authority (FNHA) (First Nations Health Authority, 2021). The FNHA is the data steward for the BC arm of the First Nations Regional Health Survey – Phase 3 (FNRHS-3), which is used in this analysis (First Nations Health Authority, 2019). The FNHA theorized an index of cultural wellness using FNRHS data that included variables for language knowledge, use of traditional food, use of traditional medicine, participation in cultural events, and valuing traditional spirituality (First Nations Health Authority, 2021). Blending this approach with our theoretical framework, reclamation of Indigenous identity, and other promoters of cultural wellness can thus be theorized to have a positive causal effect on the construct of cultural wellness. Cultural wellness in our framework is intermediate between reclamation of Indigenous identity and other promoters of cultural wellness, and holistic measures of physical, mental, emotional, and spiritual balance.

Both the theoretical construction proposed by the FNHA and the theoretical construction used in this analysis (in which multiple cultural activities are causally downstream from reclamation of Indigenous identity and other promoters of cultural wellness, as well as being indicators of a common latent construct of cultural wellness) lead to an expectation of positive associations between the multiple measures of cultural activities. One consequence of this positive association is that to identify specifically the independent association of language knowledge with measures of holistic wellness, control for confounding either by reclamation, other promoters of cultural wellness, or by measures of other cultural activities is necessary. Without controlling for these variables, language knowledge may act in models as a proxy for reclamation of both Indigenous identity and cultural wellness.

## Objectives

This study presents a closer examination of the relationship between language and wellness in First Nations populations living on reserve in British Columbia. We used a two-pronged approach that first explored Indigenous language learners experiences (Berry, 2022), and then used these qualitative results to inform our regression model used to analyze associations between language and wellness. Our first objective was to learn about the lived experiences of Indigenous language learners to inform the specification and interpretation of our models by deepening our conceptual understandings of constructs of Indigenous language fluency. The second objective was to estimate the associations between Indigenous language fluency and holistic wellness outcomes with control for potential confounders using FNRHS3 data.

## Methods

### Study design

We used results from our qualitative study with Indigenous language learners to inform our variable construction and directed acyclic graph. The results of our thematic analysis of interviews with Indigenous language learners were consistent with literature on Indigenous language learning and knowledge, which emphasized the importance of the domains of speaking and understanding an Indigenous language (Berry, 2022; Ignace, 2016; T. L. McCarty et al., 2018; Norris, 2006). Specifically, results indicated that spoken language use especially provided language learners with a deepened sense of connection to their Indigenous identity and improved wellness. We translated the importance of these results to our quantitative study by using self-reported abilities of speaking and understanding spoken Indigenous language to construct our measure of Indigenous language fluency as described below under variables. We did not include self-reported ability to read and write an Indigenous language in our measure of First Nations language fluency because, although important, previous literature supports the specific centrality of speaking and listening to language revitalization, communicative competence, and cultural continuity (Ignace, 1998, 2016).

We used cross-sectional data from the BC portion of the FNRHS3 collected between March 2015 and March 2017. The survey was funded by the First Nations Inuit Health branch of Health Canada, coordinated nationally by the First Nations Information Governance Centre, and implemented in British Columbia by the FNHA, which also served as the steward for the data repository from which our data were retrieved (First Nations Information Governance Centre, 2018). At the national level, FNRHS3 sampling was designed to represent the target population of First Nations children, youth, and adults living on First Nations reserves and in Northern communities across Canada (First Nations Information Governance Centre, 2018). The target population for our analysis only included adults aged 18 and older living on First Nations reserves in BC. In stage 1 of sampling in BC, First Nations reserves were divided into five regions. In three of the five regions, all reserves were invited to participate regardless of population size. In the other two, only reserves with a population size of at least 75 individuals were invited due to cost and logistics constraints. In stage 2, individuals were randomly selected within the 122 participating reserves using community membership lists. If an invited individual did not participate, another individual of the same group defined by age and sex was randomly invited as a replacement. Sampling weights were derived by the FNRHS3 team to enable estimation of model parameters at the BC level normalized to population distributions of age and sex (First Nations Health Authority, 2019). At the provincial level, the FNRHS3 had a community response rate of 80% and a sampling ratio of 85% (First Nations Health Authority, 2019). We obtained ethics approvals from the FNHA Data Secretariat and the Simon Fraser University Research Ethics Board via Research Ethics BC. In accordance with the principle that First Nations Peoples should maintain ownership, control, access, and possession of their research data (OCAP, a registered trademark of the First Nations Information Governance Centre), the data we used cannot be provided in a public repository.

### Variables

The exposure variable, language fluency, was defined using self-reported answers to two questions: “How well can you understand your First Nations language?” and “How well can you speak your First Nations language?” Possible responses to both questions included (1) cannot understand/speak, (2) a few words, (3) basic, (4) intermediate, or (5) fluent. We derived a three-level categorical language fluency variable with levels defined as (1) none or a few words (cannot understand/speak or a few words for both speaking and understanding), (2) basic fluency (in speaking and/or understanding and not intermediate or fluent in either), and (3) intermediate or fluent (in speaking and/or understanding). The outcome variables were feeling in balance (1) spiritually, (2) mentally, (3) emotionally, and (4) physically. We defined two-level categorical variables for each using self-reported answers to the question “How often do you feel that you are in balance in the four aspects of your life?” Respondents reported this for each domain of balance. We defined one level of our outcome variables as (1) all or most of the time; and the other level as (2) some, almost none, or none of the time. As shown in Fig. 1, we used a directed acyclic graph (Hernán & Robins, 2020; Pearl et al., 2016) and Dagitty software (Textor et al., 2017) to determine model specification to control for confounding in the association of language fluency with the four domains of feeling in balance. The definitions of the variables we included in our models for control of confounding are provided in supplementary data. We considered the node of colonialism in our DAG to be blocked due to no variability across the population in exposure to colonialism.

### Analysis

As shown in supplementary Fig. 1, there were 3026 participants aged 18 and older in the FNRHS 3. We excluded 88 individuals from all models who did not respond to questions about language fluency and an additional 312 who had missing responses for outcome and/or confounding variables. Our final sample size was 2626 individuals.

We first showed the distributions of all variables used in our analysis. We then stratified the outcome variables and covariates by levels of our exposure variable. We next used logistic regression to individually model the relative odds of each domain of balance by language fluency with and without adjustment for (1) age and sex only; and (2) age, sex plus multiple measures of participation in cultural activities. We used sampling weights provided with the FNRHS 3 data and the Complex Samples Logistic Regression procedure in SPSS Version 28 to account for the two-stage sampling scheme used in the survey.

## Results

The distributions of the variables used in our analysis are shown in Table 1. Among the 3026 adults who were included in the FNRHS3 (less missing data for each variable), 51% reported having ability with none or a few words of their First Nations language, 28% reported basic fluency, and 22% reported intermediate or fluent speaking and/or understanding. For feeling in balance all or most of the time within the four domains, the percentages of adults reporting this were 67% for physical, 72% for mental, 68% for emotional, and 70% for spiritual.

**Table 1:** Distribution of variables used in analysis among First Nation individuals aged 18 years or older living on reserve in British Columbia, Canada. First Nations Regional Health Survey—Phase 3, 2015–2017

Variable name	*n* (%)
First Nations language knowledge	
None or a few words	1143 (51%)
Basic	633 (28%)
Intermediate or fluent	696 (22%)
Feel in balance spiritually	
None, almost none, or some of the time	840 (30%)
Most or all of the time	2064 (70%)
Feel in balance mentally	
None, almost none, or some of the time	798 (28%)
Most or all of the time	2123 (72%)
Feel in balance emotionally	
None, almost none, or some of the time	924 (32%)
Most or all of the time	2022 (68%)
Feel in balance physically	
None, almost none, or some of the time	998 (33%)
Most or all of the time	1947 (67%)
Participate in community’s cultural events	
Never	309 (9%)
Rarely or sometimes	1935 (68%)
Almost always or always	754 (23%)
Sense of belonging to community	
Very or somewhat weak	483 (17%)
Somewhat or very strong	2374 (84%)
Use of traditional medicines in past 12 months	
No	1615 (57%)
Yes	1368 (43%)
Berry picking/food gathering in past 3 months	
No	2346 (76%)
Yes	634 (24%)
Fishing in the past 3 months	
No	2400 (77%)
Yes	580 (23%)
Hunting and/or trapping in past 3 months	
No	2527 (83%)
Yes	453 (17%)
Age (integer)	
18 to 30	497 (23%)
31 to 40	430 (20%)
41 to 50	394 (18%)
51 to 65	1056 (26%)
66 or older	649 (13%)
Biological Sex	
Male	1400 (48%)
Female	1538 (52%)

The distribution of variables stratified by language fluency is shown in Table 2 among the 2938 adults who reported their First Nations language knowledge (fewer missing data for each variable).

**Table 2:** Distribution of variables used in analysis stratified by degree of language knowledge among First Nations individuals aged 18 years or older living on reserve in British Columbia, Canada. First Nations Regional Health Survey – Phase 3, 2015–2017

Outcome variable		Degree of language knowledge					
	*N*	None/few words of fluency		Basic fluency		Intermediate or full fluency	
		*n*	%	*n*	%	*n*	%
Use of traditional medicine	2904	1586		624		694	
Yes		581	36.6	362	58	399	57.5
Gathering food	2898	1584		625		689	
Yes		242	15.3	188	30.1	189	27.4
Hunting or trapping	2898	1584		625		689	
Yes		196	12.4	107	17.1	134	19.4
Fishing	2898	1584		625		689	
Yes		267	16.9	153	24.5	134	19.4
Participating in community events	2915	1591		630		694	
Never		202	12.7	38	6	64	9.2
Rarely/sometimes		1118	70.3	406	64.4	344	49.6
Almost always/always		271	17.1	186	29.5	286	41.2
Sense of belonging to local community is	2787	1515		605		667	
Somewhat strong/strong		1231	81.3	502	83	588	88.2
Age	2938	1609		633		696	
18–30		312	19.4	114	18	23	3.3
31–40		275	17.1	105	16.6	30	4.3
41–50		231	14.4	98	15.5	53	7.6
51–64		560	34.8	227	35.9	263	37.8
65–97		231	14.4	89	14.1	327	47
Age	2938	1609		633		696	
18–35		441	27.4	175	27.6	39	5.6
36–55		523	32.5	206	32.5	113	16.2
56–97		645	40.1	113	39.8	544	78.2
Gender	2938	1609		633		696	
Male		790	49.1	283	44.7	327	47
Female		819	50.9	350	55.3	369	53

Results of regression modeling are shown in Tables 3, 4, 5, and 6 among 2626 adults with non-missing data needed for every model. In crude models and compared to those with none or a few words, among those with intermediate or fluent Indigenous language ability, the odds ratios (95% CI) of being in balance most or all of the time were 1.03 (0.79, 1.34) for physical balance, 1.30 (1.00, 1.69) for mental balance, 1.29 (1.00, 1.65) for emotional balance, and 1.83 (1.38, 2.41) for spiritual balance.

**Table 3:** Relative odds of feeling in balance spiritually all or most of the time by fluency of speaking and understanding one’s First Nation language among First Nations individuals aged 18 years and older in BC. First Nations RHS—Phase 3, 2015–2017

		Crude		Adjusted*		Adjusted**	
	*n*	OR (95% CI)	*p*	OR (95% CI)	*p*	OR (95% CI)	*p*
Language fluency							
None/few words	1411	Ref		Ref		Ref	
Basic	571	1.26 (0.96, 1.66)	0.094	1.29 (0.98, 1.69)	0.068	1.06 (0.82, 1.43)	0.560
Intermediate/fluent	644	1.83 (1.38, 2.41)	< 0.001	1.57 (1.18, 2.10)	0.003	1.13 (0.82, 1.55)	0.458

All* p*-values are two sided.^*^Adjusted for age and biological sex.^**^Adjusted for age, use of traditional medicine, gathering of traditional food, fishing, hunting and/or trapping, sense of belonging to community, participation in community events, and biological sex

**Table 4:** Relative odds of feeling in balance mentally all or most of the time by fluency of speaking and understanding one’s First Nation language among First Nations individuals aged 18 years and older in BC. First Nations RHS—Phase 3, 2015–2017

		Crude		Adjusted*		Adjusted**	
	*n*	OR (95% CI)	*p*	OR (95% CI)	*p*	OR (95% CI)	*p*
Language fluency							
None/few words	1411	Ref		Ref		Ref	
Basic	571	1.09 (0.84, 1.41)	0.516	1.21 (0.87, 1.45)	0.380	1.05 (0.80, 1.37)	0.732
Intermediate/fluent	644	1.30 (1.00, 1.69)	0.048	1.23 (0.93, 1.62)	0.139	1.05 (0.79, 1.41)	0.729

All *p*-values are two sided. ^*^Adjusted for age. ^**^Adjusted for age, use of traditional medicine, gathering of traditional food, fishing, hunting and/or trapping, sense of belonging to community, participation in community events, and biological sex

**Table 5:** Relative odds of feeling in balance emotionally all or most of the time by fluency of speaking and understanding one’s First Nation language among First Nations individuals aged 18 years and older in BC. First Nations RHS—Phase 3, 2015–2017

		Crude		Adjusted*		Adjusted**	
	*n*	OR (95% CI)	*p*	OR (95% CI)	*p*	OR (95% CI)	*p*
Language fluency							
None/few words	1411	Ref		Ref		Ref	
Basic	571	1.12 (0.85, 1.49)	0.420	1.15 (0.87, 1.52)	0.313	1.08 (0.81, 1.43)	0.597
Intermediate/fluent	644	1.29 (1.00, 1.65)	0.049	1.19 (0.90, 1.58)	0.217	0.99 (0.73, 1.33)	0.929

All* p*-values are two sided. ^*^Adjusted for age and biological sex. ^**^Adjusted for age, use of traditional medicine, gathering of traditional food, fishing, hunting and/or trapping, sense of belonging to community, participation in community events, and biological sex

**Table 6:** Relative odds of feeling in balance physically all or most of the time by fluency of speaking and understanding one’s First Nation language among First Nations individuals aged 18 years and older in BC. First Nations RHS—Phase 3, 2015–2017

Crude		Adjusted*	Adjusted**				
	*n*	OR (95% CI)	*p*	OR (95% CI)	*p*	OR (95% CI)	*p*
Language fluency							
None/few words	1411	Ref	Ref	Ref			
Basic	571	1.09 (0.84, 1.43)	0.365	1.16 (0.89, 1.51)	0.261	1.12 (0.86, 1.46)	0.407
Intermediate/fluent	644	1.03 (0.79, 1.34)	0.544	1.06 (0.79, 1.42)	0.691	0.94 (0.69, 1.28)	0.696

All *p*-values are two sided. ^*^Adjusted for age and biological sex. ^**^Adjusted for age, use of traditional medicine, gathering of traditional food, fishing, hunting and/or trapping, sense of belonging to community, participation in community events, and biological sex

In models adjusted for age and biological sex, these were 1.06 (0.79, 1.42) for physical balance, 1.23 (0.93, 1.62) for mental balance, 1.19 (0.90, 1.58) for emotional balance, and 1.57 (1.18, 2.10) for spiritual balance. In models adjusted for age, sex and multiple cultural activities, these were 0.94 (0.69, 1.28) for physical balance; 1.05 (0.79, 1.41) for mental balance, 0.99 (0.73, 1.33) for emotional balance, and 1.13 (0.82, 1.55) for spiritual balance.

## Discussion

We did not observe statistically significant independent associations of language fluency with any of four domains of wellness in fully adjusted models. However, our results are consistent with the hypothesis that First Nations cultural activities promote mental, emotional, and spiritual balance among adults living on reserve in what is known as British Columbia, Canada. We reach this interpretation from our models adjusted only for age and sex. Our model on spiritual balance provides the most unambiguous support for this interpretation. In that model, compared to those with none or a few words, the relative odds (95% CI) of feeling in spiritual balance most or all of the time were 1.29 (0.98, 1.69) for those with basic speaking and/or verbal understanding and 1.57 (1.18, 2.10) for those with fluent speaking and/or verbal understanding. The associations were monotonically increasing and the odds ratios for the more extreme contrast were greater than 1 throughout the range of the 95% CI. Although this was true also for the crude models for mental and emotional balance, it was no longer the case in these models after adjustment for age and sex. Thus, confounding by age and sex is a plausible explanation for the more unambiguous nature of these two crude modeling results.

Our interpretation is consistent with the hypothesis that First Nations cultural activities including language, rather than Indigenous language fluency specifically, promote spiritual balance. We reach this interpretation by comparing the results of our model adjusted only for age and sex to that adjusted for age, sex, and other cultural activities. In the latter model, compared to those with none or a few words, the relative odds (95% CI) of feeling in spiritual balance most or all of the time were 1.06 (0.82, 1.43) for those with basic speaking and/or verbal understanding and 1.13 (0.82, 1.55) for those with fluent speaking and/or verbal understanding. The addition of multiple cultural activities diminished the magnitudes of association that we saw in the model adjusted only for age and sex. The associations in the more adjusted model are still monotonically increasing, but the odds ratios are no longer consistently greater than 1 throughout the range of the 95% CI. Thus, confounding by reclamation and other upstream promoters of cultural wellness, and/or multiple cultural activities is a plausible explanation for the results we found in the model adjusted only for age and sex, which leads to our interpretation. The age- and sex-adjusted models could be confounded by multiple cultural activities if our DAG is correct and/or if multiple cultural activities (including language fluency) are indicators of a common construct of cultural wellness as discussed above. These two hypotheses are not inconsistent with each other. One might theorize that reclamation of Indigenous identity promotes cultural wellness (a latent construct indicated by participation in cultural activities) (First Nations Health Authority, 2021) which is intermediate between reclamation of Indigenous identity and spiritual balance. Other promoters of cultural wellness that are not adjusted in our models would have similar confounding effects. Our results provide promising preliminary evidence of the positive effect language and cultural activities have on wellness and provide areas of focus for future research. For example, future studies could build on evidence provided here and in other works that suggest positive effects of cultural engagement on outcomes of child and youth mental wellness, identity formation, and resilience (Barker et al., 2017; Chandler & Lalonde, 1998; Hallett et al., 2007; Kirmayer et al., 2009).

One limitation to our interpretation is the precision of estimation in our models. The 95% CI for the relative odds of spiritual balance all or most of the time among those with fluent speaking and/or verbal understanding compared to those with none or a few words was 1.18 to 2.10. If we interpret this as the range of estimated associations that are compatible with our data at alpha = 0.05 (Amrhein et al., 2019), then our data are compatible with very small magnitudes of association greater than but near 1.18. Although it would still be correct in this case to interpret that cultural wellness or participation in cultural activities promotes spiritual balance in this population, such small magnitudes of association might not make it a major contributor to spiritual balance. On the other hand, magnitudes of association near or at OR = 2.10 are also compatible with our data, and such associations would suggest that cultural wellness or participation in cultural activities might be important promoters of spiritual balance. This ambiguity of interpretation might be reduced by increasing the sample size of the analysis and thus the precision of model parameter estimation. One way to do this in future analyses would be to pool the data we used with the data from previous phases of the FNRHS.

Additional limitations of our analysis were construct validity and potential measurement error in both our exposure and outcome variables. Indigenous language revitalists and Western fluency scales measure and report fluency differently (Ignace, 2016; Norris, 2006). Measures of cultural and communicative competence, such as being able to appropriately address Elders, are indicative of valuable fluency and knowledge. Abuse perpetrated within residential schools has also led to ‘silent speakers’, who are people with a high degree of language knowledge but who are unable to speak the language. The fluency measures available in the FNRHS-3 may have inaccurately measured the language ability of silent speakers, and self-reports of fluency may be influenced by factors potentially associated with wellness outcomes, such as the fluency of other speakers in a community (Ignace, 1998). Reading and writing fluency were not included in the analysis, and the associations of these factors with the outcomes are unknown. Self-report of both exposure and outcome variables could have led to information bias.

Multiple qualitative studies demonstrate the healing benefits of language, connected to culture and identity, across many domains of wellness (Chew et al., 2015; Hallett et al., 2007; Lee, 2009; T. L. McCarty et al., 2018; Oster et al., 2014; Taff et al., 2018; Whalen et al., 2016). These studies often emphasize, either in their methodology or within the results, the inextricable link between language, culture, and the spirit of language. As persons more deeply engage with language learning, which we have framed as a significant component of reclamation of identity among other promoters of cultural wellness, deeper connections are made between wellness, identity, and spirituality. Our theoretical framework aligns with the results suggested by these other studies.

Some of our results differ from other studies which demonstrated less ambiguous beneficial effects of language and culture on wellness outcomes. A mixed-methods study with First Nations in Alberta (Oster et al., 2014) which contextualized language knowledge as a form of cultural continuity demonstrated that communities with a higher prevalence of language knowledge had significantly lower prevalence of diabetes in simple and multiple regression models (*β* = − 0.973, *t* = − 2.96, *p* = 0.007). Chandler, Hallett, and Lalonde’s work demonstrated over multiple studies the protective effect of cultural continuity (Chandler & Lalonde, 1998), and then Indigenous language (Hallett et al., 2007; Taff et al., 2018) on rates of youth suicide on reserve. Nonetheless, although some of our models had more ambiguous results, due to the width of our confidence intervals, we also demonstrated that our data were not entirely inconsistent with these previous studies.

Future research could utilize the FNHA cultural wellness index (First Nations Health Authority, 2021) to model the causal effects of participation more directly in cultural activities on health and wellness outcomes. This index could also be expanded to include additional cultural activities to measure the generalized wellness promotion effect of cultural activities more precisely. Additional research that expands upon other causal precursors to language knowledge and wellness, such as early exposure to the language or self-motivation to learn an Indigenous language, could benefit our understanding of what factors may support Indigenous Peoples re-learning their languages.

## Conclusion

We did not observe statistically significant, independent associations of language fluency with any of four domains of wellness in fully adjusted models. However, our results are consistent with the hypothesis that participation in cultural activities promotes spiritual balance among adults living on First Nations reserves in British Columbia, Canada.

### Contributions to knowledge

What does this study add to existing knowledge?

- Our analysis applied directed acyclic graphs within a theoretical framework that includes culture and reclamation as part of First Nations wellness. This is a novel application of directed acyclic graphs.
- We found a significant positive association between language knowledge and spiritual balance among First Nations adults living on reserve, adjusted for age and sex.
- Other statistical analyses completed indicate that multiple indicators of cultural engagement, rather than language knowledge independently, may positively support wellness in the four aspects.

What are the key implications for public health interventions, practice, or policy?

- Our findings suggest that policies, practices, and interventions that support the maintenance and revitalization of First Nations languages could have a positive impact on the health and well-being of First Nations adults in multiple domains. This finding is aligned with other literature and knowledge that identify language as a significant determinant of health and identity.
- Additionally, our findings suggest that multiple cultural activities have benefits for health and well-being. This supports policies and programmes that engage with a wide variety of cultural activities, rather than a single activity being the most beneficial.

## Supplementary Information

Below is the link to the electronic supplementary material. 

ESM1 Supplementary File 1 Description: A text document which describes how variables used in the statistical analyses were created. (DOCX 14.4 KB) 

ESM2 Supplementary File 2 Description: An image of a flowchart which shows how many participants were included and excluded at each step to reach the final number of participants included in the analysis. (DOCX 11.2 KB)

## Data Availability

The data underlying this article cannot be shared due to data access control processes within the First Nations Health Authority and the sensitivity of some data. This data is stewarded by the First Nations Health Authority, and all data access requests must be done through direct communication with the FNHA.

## Code availability

Not applicable per the above data access protocol.
