# Supplementary material for: Associations of Indigenous language knowledge and physical, emotional, mental, and spiritual balance among First Nations living on reserve in British Columbia, Canada
Source: Can J Public Health. 2025 Jul 1;117(2):251–60. doi: 10.17269/s41997-025-01077-7 (PMC13076809; doi:10.17269/s41997-025-01077-7)
Supplement: Supplementary file 1 — Description: A text document which describes how variables used in the statistical analyses were created. (DOCX 14.4 KB) [file 41997_2025_1077_MOESM1_ESM.docx]

Supplementary File 1

Article: Associations of Indigenous language knowledge and physical, emotional, mental, and spiritual balance among First Nations living on reserve in British Columbia, Canada

Journal: Canadian Journal of Public Health

Description of control variables used in analyses

Variables included in models for control of confounding were 1) age, 2) sense of belonging to local First Nations community, 3) participation in community events, 4) fishing (in the past 3 months, 5) gathering traditional foods (in the past 3 months), 6) the use of traditional medicine (in the past 12 months), and 7) hunting or trapping (in the past 3 months). Each variable was derived from a person’s self-reported answers to questions the First Nations Regional Health Survey – Phase 3.

The variable for age was defined using answers to the question “What is your age?”. Answers were recorded in whole years as a continuous variable. We derived a five-level categorical variable with levels defined as 1) 18-30, 2) 31-40, 3) 41-50, 4) 51-65, 5) 65-97 years. We chose to define the first age group as such to reflect how First Nations conceptualize ‘youth’.

The variable for gender was defined using answers to the question “What is your gender?”. Answers were recorded as male or female, with no option for participants to indicate non-binary genders, two-spirit identity, or if they were transgender. We derived a binary variable for gender.

The variable for sense of belonging was defined using self-reported answers to the question “How would you describe your sense of 1 belonging to your local community? Would you say it is?”. Possible responses included 1) very strong, 2) somewhat strong, 3) somewhat weak, and 4) very weak. We derived a two-level categorical variable with levels defined as 1) very strong or somewhat strong and 2) somewhat weak or very weak.

The variable for participation in events was defined using answers to the question “How often do you participate in community events?” with responses including 1) never, 2) rarely, 3) sometimes, 4) almost always, and 5) always. We derived a three-level categorical variable with levels of 1) never, 2) rarely/sometimes and 3) almost/always or always.

The variables for fishing, gathering, and use of traditional medicine were derived from responses to the question 1) “Have you done any of the following activities in the past 3 months? Fishing”, 2) “Have you done any of the following activities in the past 3 months? Berry picking or other food gathering” Respondents were able to choose multiple activities from a list that included 1) fishing, 2) Berry picking or other food gathering, and 3) “In the past 12 months, did you used traditional medicine? Note: Traditional medicine can include herbal remedies, spirit”. We derived a binary categorical variable for each question coded as ‘yes’ or ‘no’.

The variable for hunting and/or trapping was defined using answers to two questions: 1) “Have you done any of the following activities in the past 3 months? Hunting”, and 2) “Have you done any of the following activities in the past 3 months? Trapping”. Possible answers included “yes” or “no”. There were insufficient responses to separate hunting and trapping into individual covariates, so they were combined to create one binary categorical variable with levels of 1) Neither hunting nor trapping in the past 3 months or 2) Hunting and/or trapping in the past 3 months.
