# Supplementary material for: Associations of Indigenous language knowledge and physical, emotional, mental, and spiritual balance among First Nations living on reserve in British Columbia, Canada
Source: Can J Public Health. 2025 Jul 1;117(2):251–60. doi: 10.17269/s41997-025-01077-7 (PMC13076809; doi:10.17269/s41997-025-01077-7)
Supplement: Supplementary file 2 — Description: An image of a flowchart which shows how many participants were included and excluded at each step to reach the final number of participants included in the analysis. (DOCX 11.2 KB) [file 41997_2025_1077_MOESM2_ESM.docx]

Participants in the study (n=3026)

Participants who did not answer questions about language knowledge (n=88)

Participants who answered questions about language knowledge (n=2938)

Participants with missing outcome and covariate data (n=312)

Participants included in analysis (n=2626)
